# Promoting Positive Nursing Practice Environments: Outcomes of a Randomized Trial in Primary Care

**DOI:** 10.1155/jonm/4139767

**Published:** 2026-03-15

**Authors:** Soraia Cristina de Abreu Pereira, Eduardo José Ferreira dos Santos, Rosilene Alves Ferreira, Cintia Silva Fassarella, Olga Maria Pimenta Lopes Ribeiro

**Affiliations:** ^1^ School of Medicine and Biomedical Sciences, University of Porto, Porto, Portugal, up.pt; ^2^ RISE-Health, Porto, Portugal; ^3^ Nursing School, University of Porto, Porto, Portugal, up.pt; ^4^ Higher School of Health, Polytechnic Institute of Viseu, Viseu, Portugal, ipv.pt; ^5^ Health Sciences Research Unit-Nursing (UICISA: E), Coimbra, Portugal; ^6^ Portugal Centre for Evidence Based Practice, Coimbra, Portugal; ^7^ Faculty of Nursing, The State University of Rio de Janeiro, Rio de Janeiro, Brazil

**Keywords:** nurses, patient safety, primary health care, randomized controlled trial, working conditions

## Abstract

**Background:**

The nursing practice environment is widely recognized as a key factor in promoting care quality and safety. It is associated with greater professional satisfaction, improved nurse retention, and reduced adverse events. Despite its importance being broadly acknowledged in the literature, there are few structured interventions aimed at promoting the nursing practice environment.

**Objective:**

To evaluate the effectiveness of the Positive Nursing Practice Environment Promotion Program (PPAPEP) in improving primary care nurses’ perceptions of their work environment and attitudes toward patient safety.

**Methods:**

A randomized, controlled, parallel‐group, double‐blind clinical trial was conducted in primary healthcare units in Northern Portugal between September 2024 and April 2025. A total of 48 nurses participated, allocated by stratified randomization into intervention and control groups. The instruments Scale for the Environments Evaluation of Professional Nursing Practice‐Shortened Version and the Safety Attitudes Questionnaire‐Short Form were applied at three time points: preintervention, postintervention, and three‐month follow‐up. Statistical analysis included paired and independent *t*‐tests, repeated‐measures ANOVA, and correlation analysis.

**Results:**

The PPAPEP produced modest improvements in overall perceptions of the nursing practice environment in the intervention group, with small significative changes in the structure and process subscales. No statistically significant changes were observed in the safety climate. Follow‐up analyses showed an inverted U‐shaped pattern, indicating that the immediate positive effects slightly decreased over time. Perceptions of the practice environment and safety climate were strongly correlated at all time points.

**Conclusions:**

The PPAPEP has demonstrated effectiveness in improving the nursing practice environment. Effects on the safety climate were limited, suggesting that sustained changes require continuous interventions, periodic reinforcement, and institutional involvement. The strong association between nurses’ perceptions of the work environment and the safety climate highlights the need for integrated strategies that simultaneously promote good working conditions and patient safety.

**Trial Registration:** ClinicalTrials.gov.identifier: NCT06762015

## 1. Introduction

In recent decades, the nursing practice environment has emerged as a key factor in promoting the quality and safety of healthcare. There is a growing body of evidence showing that positive nursing practice environments are associated with multiple benefits, such as increased staff satisfaction, reduced burnout, and higher nurse retention rates. Most importantly, they have a significant impact on patient safety and the quality of care provided [1–4]. A positive nursing practice environment also contributes to reducing adverse events, such as falls, healthcare‐associated infections, and pressure ulcers [5–9]. It fosters a safer, more effective, and patient‐centered organizational culture. This is defined as a work environment that facilitates the delivery of high‐quality care, supports healthcare professionals, encourages their involvement in decision‐making, and promotes effective communication and collaboration [10]. The existence of sufficient resources, balanced workloads, professional recognition, and opportunities for growth are essential components of an environment that protects nurses’ well‐being and promotes more favorable clinical outcomes [11–14].

The relevance of practice environments is even more evident in the context of primary healthcare, since these health units represent the first point of contact with the healthcare system. They require continuity, proximity, and person‐centered care and are often delivered in settings facing significant organizational challenges [15–17]. Despite the recognized importance of positive environments at this level of care, there are few specific and systematized interventions aimed at promoting structured improvements in the nursing practice environment in primary healthcare [4]. The literature shows that most existing initiatives are short term and address isolated issues. Multicomponent programs that are grounded in theoretical models and scientifically validated evidence are uncommon [18–20].

In light of these gaps and the urgent need to empower nurses to improve their work environment, the Program for the Promotion of Positive Nursing Practice Environments (PPAPEP) was developed [4]. Developed from a comprehensive literature review and a concept analysis of the positive nursing practice environment, and validated by a panel of experts through an e‐Delphi study [4], it comprises six training sessions that address the factors and variables influencing the positive nursing practice environment, its attributes, and the expected outcomes of its implementation (Table 1). The PPAPEP uses an active methodology that focuses on reflection and group discussion. It aims to develop professionals’ competencies to positively transform their work environment, such as communication and collaborative work.

**TABLE 1:** Content of the PPAPEP.

Session	Content
1	Presentation of the program. Characteristics and variables related to clients and professionals that contribute to a Positive Nursing Practice Environment.
2	Characteristics and variables related to institutions that contribute to a Positive Nursing Practice Environment.
3	Attributes of a Positive Nursing Practice Environment.
4	Outcomes of a Positive Nursing Practice Environment related to professionals
5	Outcomes of a Positive Nursing Practice Environment related to institutions and clients.
6	Group dynamics to consolidate the knowledge acquired throughout the program. Conclusion of the PPAPEP

*Note:* PPAPEP—Positive Nursing Practice Environment Promotion Program.

This study presents the results of the first randomized controlled trial evaluating the effectiveness of the PPAPEP in improving primary healthcare nurses’ perceptions of safety climate and the nursing practice environment [21]. The data obtained are expected to provide robust evidence in support of organizational strategies, guide continuous improvement policies, and reinforce the importance of a positive nursing practice environment as cornerstones for a safer, more effective, and humanized healthcare system.

### 1.1. Study Aim

This study aimed to evaluate the effectiveness of the PPAPEP in improving primary care nurses’ perceptions of their work environment and attitudes toward patient safety. More specifically, the study sought to determine whether participation in the PPAPEP led to statistically significant and clinically meaningful improvements in scores on the Scale for the Environments Evaluation of Professional Nursing Practice‐Shortened Version (SEE‐NP) [22] and the Safety Attitudes Questionnaire‐Short Form (SAQ‐SF) [23], as compared to a control group (CG) that received no intervention.

## 2. Methods

### 2.1. Study Design

This study was a randomized, controlled, parallel‐group clinical trial with double‐blinding (participants and investigators). It was conducted in primary healthcare units within a local health unit in Northern Portugal between September 2024 and April 2025, with first recruitment in October 2024. The study was registered at ClinicalTrials.gov and was conducted in accordance with a previously published protocol [21].

### 2.2. Participants

Participants were nurses from primary healthcare units (e.g., family health units and primary care centers) who provided first‐contact, continuous, comprehensive, and coordinated person‐centered care. All nurses who expressed an interest in the intervention and provided informed consent were included, regardless of their academic qualifications, professional category, or experience level. Those who failed to attend all six training sessions were excluded from the final analysis.

### 2.3. Randomization

A stratified randomization procedure was used to allocate participants equally to the intervention group (IG) and CG in a 1:1 ratio. The stratification criteria included sociodemographic and professional variables such as gender, age, academic background, professional category, overall experience, primary care experience, and unit type. Randomization was executed using Random.org by an independent researcher uninvolved in the intervention or data analysis, in order to ensure allocation concealment and eliminate selection bias.

### 2.4. Ethical Considerations and Study Registration

Ethical approval for the study protocol was granted by the Health Ethics Committee (CES no. 57_2024) and the administrative board of the participating institution. The trial was also registered on ClinicalTrials.gov. All participants were given detailed information about the study’s objectives, procedures, data confidentiality, and their right to withdraw at any time without penalty. Written informed consent was obtained from all participants.

### 2.5. Sample Size Estimation

Sample size was calculated using G × Power 3.1.9.6 software for a two‐tailed paired *t*‐test, targeting an effect size of 0.5, a significance level (*α*) of 0.05, and a power of 0.80. The minimum required sample was 34 participants (17 per group).

### 2.6. Intervention

The IG participated in the PPAPEP, consisting of six weekly face‐to‐face sessions, lasting 3 hours each, for a total of 18 h. The program was validated through a Delphi study with expert consensus. The training focused on the characteristics, attributes, and outcomes of positive nursing environments and was delivered using slide presentations, videos, and group activities to encourage reflection and active participation. Each session combined brief theoretical input with participatory methodologies, including guided group discussions, reflective exercises, and small‐group activities focused on real practice scenarios. Supporting information (available here) included standardized presentation slides, session guides, and reflection prompts aligned with the program’s objectives. To ensure intervention fidelity, all sessions were delivered by the same trained facilitator using a standardized intervention manual and predefined session plans. Attendance was monitored in all sessions, and session content was delivered consistently across participating units.

### 2.7. Control

The CG completed evaluations prior to receiving the intervention. Although these participants eventually joined the PPAPEP, only the preintervention data were used for comparative purposes in this study phase.

### 2.8. Data Collection and Outcome Measurement

Data were collected at three time points: baseline (EM 1), postintervention (EM 2), and at the three‐month follow‐up (EM 3). The instruments used included the SEE‐NP: This assesses perceptions of nursing work environments across three subscales (structure, process, and outcome). Items are rated on a 5‐point Likert scale. Higher scores indicate more favorable environments. The SAQ‐SF evaluates professionals’ perceptions of patient safety climate across six dimensions. Responses are given on a 5‐point Likert scale and converted to a score between 0 and 100, with higher scores indicating a more positive safety climate.

### 2.9. Statistical Analysis Methods

The data were analyzed in accordance with the CONSORT guidelines. Descriptive statistics (means, standard deviations, frequencies, and confidence intervals) summarized the participant characteristics. Assumptions of normality, linearity, and homoscedasticity were checked prior to conducting inferential analyses. Given the sample size (*n* < 50), the Shapiro–Wilk test was used as the primary criterion to evaluate whether the data adhered to a normal distribution. Most variables exhibited a normal distribution in both the CG and the IG, with *p* values > 0.05. However, slight deviations from normality were observed for the variables “age” (*p* = 0.036) and “process subscale mean at time 0” (*p* = 0.004) in the IG. These results suggest that the assumptions required for parametric tests were generally met, enabling the use of the independent samples Student’s *t*‐test. Nevertheless, variables that did not meet the normality assumption were analyzed with caution, considering the robustness of parametric tests to moderate deviations from normality in relatively balanced samples (*n* > 15 per group). Paired and independent sample *t*‐tests were used to assess within‐ and between‐group differences. Multivariate analyses, including repeated‐measures ANOVA and linear regression, were used to explore the association of sociodemographic and professional variables on the outcomes. Effect sizes were interpreted using Cohen’s d (small = 0.2–0.49, moderate = 0.5–0.8, and large > 0.8). Corrections (e.g., Greenhouse–Geisser) were applied where the sphericity assumption was violated.

## 3. Results

### 3.1. Characteristics of the Participants

**FIGURE 1:**
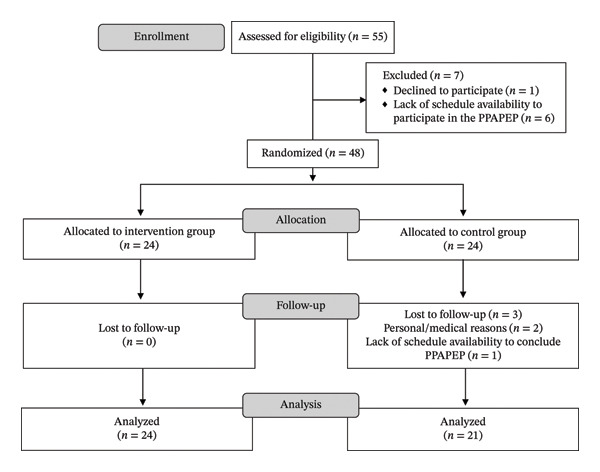
CONSORT flow diagram. Abbreviations: CONSORT—Consolidated Standards of Reporting Trials; PPAPEP—Positive Nursing Practice Environment Promotion Program.

Following the dissemination of the PPAPEP, 55 nurses expressed an interest in participating in the study. Of these, only 48 completed the baseline questionnaires, with 24 randomized to the IG and 24 to the CG. Three participants withdrew from the CG during the study (Figure 1).

The mean age of participants was 45.75 years (SD = 7.81), and the majority were female (*n* = 41; 85.4%). Regarding professional status, half of the sample were specialist nurses (*n* = 24; 50%), with a mean of 22.10 years of professional experience (SD = 7.910) and an average of 16.15 years working in primary health care (SD = 9.406). The majority held permanent contracts (*n* = 43; 89.6%) and worked in family health units (*n* = 26; 54.2%) (Table 2). No statistically significant differences were observed between IG and CG in any demographic, professional, or baseline outcome variable, including age (*p* = 0.77), years of professional experience (*p* = 0.79), years in primary health care (*p* = 0.868), or baseline scores on the SEE‐NP and SAQ‐SF instruments (data not shown). This confirmed baseline homogeneity, enabling valid group comparisons to be made.

**TABLE 2:** Demographic and professional characteristics of all participants, including those in the intervention and control groups.

Characteristics/study outcomes	Total (*N* = 48)	Groups	
		Intervention (*n* = 24)	Control (*n* = 24)
Gender, *n* (%)			
Female	41 (85.4)	20 (83.3)	21 (87.5)
Male	7 (14.6)	4 (16.4)	3 (12.5)
Age, years, mean (SD)	45.75 (7805)	46.08 (7101)	45.42 (8592)
Marital status, *n* (%)			
Single	5 (10.4)	1 (4.2)	4 (16.7)
Married/nonmarital partnership	38 (79.2)	22 (91.7)	16 (66.7)
Divorced	4 (8.3)	1 (4.2)	3 (12.5)
Widowed	1 (2.1)	0 (0)	1 (4.2)
Educational level, *n* (%)			
Bachelor’s degree	39 (81.3)	19 (79.2)	20 (83.3)
Master’s degree	8 (16.7)	5 (20.8)	3 (12.5)
Doctoral degree	1 (2.1)	0 (0)	1 (4.2)
Professional category, *n* (%)			
Nurse	23 (47.9)	12 (50)	11 (45.8)
Nurse specialist	24 (50)	12 (50)	12 (50)
Nurse manager	1 (2.1)	0 (0)	1 (4.2)
Time of professional practice, years, mean (SD)	22.10 (7910)	22.42 (7330)	21.79 (8592)
Time of professional practice in PHC, years, mean (SD)	16.15 (9406)	16.38 (9921)	15.92 (9069)
Employment contract, *n* (%)			
Fixed‐term contract	4 (8.3)	2 (8.3)	2 (8.3)
Indefinite‐term contract	1 (2.1)	0 (0)	1 (4.2)
Permanent contract	43 (89.6)	22 (91.7)	21 (87.5)
Professional practice setting, *n* (%)			
Family health units	26 (54.2)	13 (54.2)	13 (54.2)
Community care unit	8 (16.7)	4 (16.7)	4 (16.7)
Public health unit	5 (10.4)	3 (12.5)	2 (8.3)
Basic emergency service	5 (10.4)	2 (8.3)	3 (12.5)
Local coordination team/nursing directorate	4 (8.4)	2 (8.3)	2 (8.3)

Abbreviations: PHC, primary health care; SD, standard deviation.

### 3.2. Primary Results

Primary outcomes focus on participants’ perceptions of the nursing practice environment and safety climate in primary healthcare settings.

#### 3.2.1. Perception on the Nursing Practice Environment

Nurses’ perceptions of the practice environment, as measured by the SEE‐NP, in the CG showed a significant improvement in the structure subscale from pretest (*M* = 2.86, SD = 0.48) to posttest (*M* = 3.01, SD = 0.59), *t* (20) = −2.16, *p* = 0.043, with a moderate effect size (Hedges’ *d* = 0.45). No significant change was observed in the process subscale, which increased slightly from 3.57 to 3.62 (*t* (20) = −0.43, *p* = 0.670, *d* = 0.09). The outcome subscale exhibited a trend toward improvement, rising from 2.89 to 3.10 (*t* (20) = −1.96, *p* = 0.064, *d* = 0.41). Overall perception of the nursing practice environment in the CG showed a modest, nonsignificant increase from 3.09 to 3.23 (*d* = 0.34) (Table 3).

**TABLE 3:** Intervention effects on nursing practice environment and safety climate.

	Control group (*n* = 21)			Intervention group (*n* = 23)			Diff. in changes (95% CI)	*p* value
	Pre (mean ± SD)	Post (mean ± SD)	Mean change (95% CI)	Pre (mean ± SD)	Post (mean ± SD)	Mean change (95% CI)		
SEE‐NP structure subscale	2.86 ± 0.48	3.01 ± 0.59	−0.15 (−0.30; −0.01)	3.09 ± 0.67	3.08 ± 0.47	+0.00 (−0.20; +0.21)	0.16 (−0.22; 0.54)	0.416
SEE‐NP process subscale	3.57 ± 0.70	3.62 ± 0.74	−0.06 (−0.32; +0.21)	3.91 ± 0.56	4.02 ± 0.58	−0.11 (−0.34; +0.12)	0.28 (−0.13; 0.68)	0.179
SEE‐NP outcome subscale	2.89 ± 0.81	3.10 ± 0.90	−0.22 (−0.45; +0.01)	2.96 ± 0.82	3.05 ± 0.83	−0.09 (−0.41; +0.23)	−0.05 (−0.58; 0.47)	0.842
SEE‐NP	3.09 ± 0.53	3.23 ± 0.62	−0.13 (−0.30; +0.04)	3.33 ± 0.60	3.52 ± 0.55	−0.19 (−0.40; +0.02)	0.29 (−0.07; 0.65)	0.109
SAQ‐SF	3.42 ± 0.55	3.57 ± 0.53	−0.15 (−0.29; −0.01)	3.57 ± 0.52	3.51 ± 0.48	+0.06 (−0.12; +0.23)	−0.06 (−0.36; 0.25)	0.698

*Note:* SEE‐NP: Scale for the Environments Evaluation of Professional Nursing; SAQ‐SF: Safety Attitudes Questionnaire‐Short Form. Values are expressed as mean ± SD (standard deviation). Intra‐group changes from pre‐ to postintervention were assessed using paired *t*‐tests. Inter‐group differences in change scores were analyzed using independent‐samples *t*‐tests. Pre = pretest; Post = posttest; 95% CI = 95% confidence interval.

In the IG, composed of 23 participants, the SEE‐NP overall showed a trend toward significance (pre = 3.33; post = 3.52), *t* (22) = −1.85, *p* = 0.077, with a small‐to‐moderate effect (*d* = −0.37). The structure subscale remained stable, and no significant differences were found between time points (pre = 3.09; post = 3.08), *t* (22) = 0.04, *p* = 0.970, with a null effect (*d* = 0.01). The process subscale showed a small increase from 3.91 to 4.02, which was not statistically significant (*t* (22) = −0.98, *d* = 0.20). Similarly, the outcome subscale did not change significantly (2.96–3.05, *d* = 0.12). The overall perception of the nursing practice environment showed a trend toward improvement from 3.33 to 3.52, with a small‐to‐moderate effect size (*t* (22) = −1.85, *p* = 0.077, *d* = 0.37) (Table 3).

These findings suggest that although the CG experienced significant improvement in structure and trends in outcome, the IG displayed only modest, nonsignificant changes. This indicates that the intervention had a limited immediate impact of the intervention on nurses’ perceptions of the practice environment. Paired correlations were statistically significant for all variables in both groups, indicating strong associations between pre‐ and posttest scores. In the CG, correlations ranged from *r* = 0.675 to *r* = 0.841, and in the IG, from *r* = 0.571 to *r* = 0.711 (*p* < 0.01 for all pairs). In the CG, significant improvements were observed in the structure subscale and in the overall nursing practice environment scale. In the IG, no statistically significant differences were found between time points, although a trend toward improvement was identified in the SEE‐NP. Overall, effect sizes were small to moderate, indicating modest clinical effects. The high correlations between pre‐ and postintervention values suggest measurement consistency and a potential regression‐to‐the‐mean effect (data not shown).

#### 3.2.2. Perception on Safety Climate

Regarding the perceived safety climate, measured by the SAQ‐SF, a significant improvement in mean scores was found in CG (pre = 3.42; post = 3.57), *t* (20) = −2.26, *p* = 0.035, with a small‐to‐moderate effect size (*d* = −0.47). In contrast, the IG showed a slight, nonsignificant decrease in mean scores from 3.57 to 3.51 (*t* (22) = 0.64, *p* = 0.528, *d* = 0.13), indicating stability in perceptions of safety climate over the same period (Table 3).

#### 3.2.3. Follow‐Up Analysis

A repeated‐measures analysis showed an inverted U‐shaped pattern, with an immediate increase in positive perception of the nursing practice environment after the intervention (*M* = 3.55; SD = 0.53), followed by a slight decline at follow‐up (*M* = 3.43; SD = 0.58). Multivariate tests did not indicate a statistically significant difference over time (Wilks’ lambda = 0.799; *F* (2, 19) = 2.397; *p* = 0.118), but the partial effect size was moderate (*η* ^2^ = 0.201), indicating that approximately 20% of the variance was explained by temporal changes. Polynomial contrast analysis revealed a significant quadratic trend (*F* (1, 20) = 5.028; *p* = 0.036), suggesting that the intervention produced an immediate improvement followed by a partial decline, highlighting the need for reinforcement strategies to maintain positive effects over time.

Regarding perceptions of the safety climate, SAQ‐SF scores remained stable from posttest (*M* = 3.51; SD = 0.47) to follow‐up (*M* = 3.54; SD = 0.56), *t* (20) = −0.36, *p* = 0.721; *d* = 0.08, indicating maintenance of perceived safety climate in the short and medium term (data not shown).

### 3.3. Secondary Results

Pearson correlation analyses were conducted to investigate the associations between sociodemographic variables, professional characteristics, and the psychometric dimensions assessed by the SEE‐NP and the SAQ‐SF. These analyses revealed highly significant correlations between the subscales of the SEE‐NP and SAQ instruments (Table 4). The SEE‐NP showed strong correlations with the SAQ at all time points: pre: *r* = 0.729; post: *r* = 0.771; and follow‐up: *r* = 0.804, suggesting a strong association between perceptions of the environment and safety attitudes.

**TABLE 4:** Associations between sociodemographic and professional characteristics and nurses’ perceptions of the practice environment and safety climate.

		Sex	Age	Marital status	Academic qualification	Professional category	Nursing practice time	Primary care time	Employment contract type	Context	SEE‐NP Pre‐Mean	SAQ‐SF Pre‐Mean	SEE‐NP Post‐Mean	SAQ‐SF Post‐Mean	SEE‐NP FUP‐Mean	SAQ‐SF FUP‐Mean
Sex	Pearson correlation	1														
	Sig. (2‐tailed)															

Age	Pearson correlation	−0.080	1													
	Sig. (2‐tailed)	0.590														

Marital status	Pearson correlation	−0.130	0.391	1												
	Sig. (2‐tailed)	0.378	0.006													

Academic qualifications	Pearson correlation	−0.060	−0.096	0.070	1											
	Sig. (2‐tailed)	0.688	0.515	0.637												

Professional category	Pearson correlation	−0.196	0.105	0.183	0.305^∗^	1										
	Sig. (2‐tailed)	0.181	0.479	0.213	0.035											

Nursing practice time	Pearson correlation	−0.017	0.813	0.346^∗^	−0.113	0.081	1									
	Sig. (2‐tailed)	0.910	< 0.001	0.016	0.444	0.585										

Primary care time	Pearson correlation	0.138	0.419	0.332^∗^	−0.115	0.035	0.623	1								
	Sig. (2‐tailed)	0.349	0.003	0.021	0.434	0.814	< 0.001									

Employment contract type	Pearson correlation	0.137	0.039	0.226	0.152	0.060	0.074	0.080	1							
	Sig. (2‐tailed)	0.353	0.794	0.122	0.302	0.686	0.619	0.590								

Context	Pearson correlation	0.072	−0.103	0.056	−0.014	0.302^∗^	−0.195	−0.172	−0.016	1						
	Sig. (2‐tailed)	0.626	0.486	0.705	0.927	0.037	0.184	0.244	0.911							

SEE‐NP Pre‐Mean	Pearson correlation	0.068	−0.019	−0.006	−0.069	−0.188	−0.080	0.130	0.016	−0.438	1					
	Sig. (2‐tailed)	0.656	0.902	0.969	0.653	0.217	0.601	0.395	0.916	0.003						

SAQ‐SF Pre‐Mean	Pearson correlation	−0.103	−0.026	−0.061	0.018	−0.178	−0.080	0.155	0.180	−0.472	0.729	1				
	Sig. (2‐tailed)	0.501	0.866	0.691	0.908	0.242	0.600	0.308	0.237	0.001	< 0.001					

SEE‐NP Post‐Mean	Pearson correlation	0.040	−0.112	−0.103	−0.080	−0.209	−0.205	0.008	−0.094	−0.491	0.725	0.660	1			
	Sig. (2‐tailed)	0.797	0.468	0.507	0.604	0.174	0.183	0.959	0.543	< 0.001	< 0.001	< 0.001				

SAQ‐SF Post‐Mean	Pearson correlation	−0.104	−0.119	−0.271	−0.029	−0.270	−0.145	−0.054	−0.038	−0.533	0.568	0.736	0.771	1		
	Sig. (2‐tailed)	0.503	0.442	0.075	0.853	0.076	0.346	0.730	0.806	< 0.001	< 0.001	< 0.001	< 0.001			

SEE‐NP FUP‐Mean	Pearson correlation	0.279	−0.166	−0.018	0.059	−0.282	−0.165	0.014	0.157	−0.616	0.709	0.632	0.840	0.808	1	
	Sig. (2‐tailed)	0.209	0.460	0.937	0.794	0.204	0.464	0.951	0.486	0.002	< 0.001	0.002	< 0.001	< 0.001		

SAQ‐SF FUP‐Mean	Pearson correlation	0.045	−0.130	0.117	0.082	−0.332	−0.132	0.029	0.103	−0.494^∗^	0.638	0.700	0.665	0.714	0.804	1
	Sig. (2‐tailed)	0.843	0.563	0.605	0.715	0.131	0.557	0.899	0.647	0.019	0.001	< 0.001	0.001	< 0.001	< 0.001	

*Note:* The sample (*N*) was 48 for all variables, except for SEE‐NP Pre‐Mean and SAQ‐SF Pre‐Mean with 45, SEE‐NP Post‐Mean and SAQ‐SF Post‐Mean with 44, and finally, SEE‐NP FUP‐Mean and SAQ‐SF FUP‐Mean with 22.

^∗^Correlation is significant at the 0.05 level (2‐tailed).

Although professional practice duration was found to be correlated with time in primary health care (*r* = 0.623, *p* < 0.001), no significant correlations were observed with the evaluated scale. This suggests that despite accumulated experience, perceptions of the professional environment and safety attitudes are not directly modified by professional experience.

The work context showed negative and significant correlations with several variables, such as SEE‐NP (Pre): *r* = −0.438, *p* = 0.003 and SAQ (Pre): *r* = −0.472, *p* = 0.001, suggesting that professionals in certain contexts perceive the environment and safety attitudes more negatively. An impact of context on safety attitudes was also observed: SAQ Pre: *r* = −0.472, *p* = 0.001; SAQ Post: *r* = −0.533, *p* < 0.001; SAQ follow‐up: *r* = −0.494, *p* = 0.019. Once again, less favorable contexts were associated with more negative attitudes toward safety. Professional category was found to correlate negatively with both the SAQ‐SF and SEE‐NP at different time points. This suggests that professionals in higher‐ranking or specialized positions may hold a more critical view of the environment and express lower satisfaction with the safety culture.

Sociodemographic variables such as age, sex, marital status, academic qualifications, and professional variables such as years of service, primary health care practice duration, and employment contract type did not show significant correlations with either the SEE‐NP or the SAQ‐SF, suggesting that neither tenure within the institution nor type of contract has a substantial influence on perceptions of the environment or significantly affects safety attitudes.

## 4. Discussion

This study evaluated the impact of the PPAPEP on nurses’ perceptions of the nursing practice environment and the perceived safety climate in primary healthcare, providing insights into the potential benefits and limitations of this intervention aimed at improving the nursing practice environment.

The intervention produced modest improvements in nurses’ perceptions of the practice environment, as assessed by the SEE‐NP. The IG showed an increase in the overall SEE‐NP score after the intervention, reflecting a small to moderate effect size and a trend toward statistical significance. At the same time, the improvements observed in the CG, particularly about certain selected dimensions of the practice environment and the safety climate, require careful interpretation. These changes may reflect contextual organizational dynamics unrelated to the intervention itself, such as ongoing institutional initiatives, baseline organizational maturity, or increased awareness resulting from study participation. Furthermore, the actual presence of the Hawthorne effect cannot be excluded with certainty, since participants, knowing that they are being observed or studied through their participation in the assessment process, may have increased their reflection and attention to safety‐related practices among professionals in the CG. This could lead to some unexplained level of performance bias. Even so, these findings highlight the complexity of evaluating organizational interventions and reinforce the importance of considering contextual and temporal factors when interpreting results.

To further explore these findings, subscale analyses revealed stability in the structure subscale of the SEE‐NP, small increases in the process subscale, and limited changes in the outcome subscale. The structure subscale comprises four dimensions: institutional policies and nurses’ involvement, people management and leadership in the service, care organization, and issues related to the physical environment and conditions for nursing practice [22]. In the context of PPAPEP implementation, the program addressed the need for nurse involvement, people and talent management, and managerial support. However, the findings indicate stability in the structure subscale, suggesting that PPAPEP faced difficulties in promoting significant structural changes. This may be due to the challenges involved in modifying the physical and structural conditions of healthcare units, as these require investment and institutional planning, which are often beyond the reach of nurses. Furthermore, the near absence of nurse managers interested in participating in the program may have limited not only the implementation of improvement and support strategies but also signaled to other nurses that this topic was not prioritized by management, potentially influencing their perceptions. These findings are consistent with those of Mabona et al. [19] and Paguio et al. [20]. They highlighted that creating a favorable nursing practice environment hinges not only on changes in leadership, communication, teamwork, and promotion of professional autonomy. However, it also requires adequate resources and organizational commitment. Consequently, robust institutional support and strategic interest from leaders and managers are paramount.

The process subscale of the SEE‐NP includes three major dimensions: autonomous practice, collaborative work and continuity of care, and strategies to ensure care quality and safety. Communication and teamwork emerge as essential components [22]. PPAPEP results showed small increases in this subscale, indicating a modest but positive evolution likely related to the program’s focus on group dynamics promoting communication and strengthening teamwork. These activities aimed to encourage nurses to exchange knowledge, reflect, and coordinate their perspectives, while also building trusting working relationships. These elements are identified in the literature as fundamental for creating positive nursing practice environments [18, 19, 24]. Although the results are modest, they suggest that interventions focused on professional interaction and collaboration, even short term, can positively impact autonomous practice, continuity of care, and care quality and safety. Moreover, they reinforce the importance of structured programs prioritizing communication, teamwork, and collaborative strategies as interdependent and synergistic dimensions contributing not only to better professional outcomes but also to improved client outcomes and team satisfaction.

The outcome subscale of the SEE‐NP includes systematic evaluation of nursing care, workload, and performance indicators such as absenteeism [22]. The PPAPEP results showed limited changes in this subscale, which may be explained by the fact that aspects such as workload, absenteeism, or patient safety depend on broader structural and institutional factors that are beyond the program’s direct influence. However, the literature shows that interventions focused on leadership, communication, teamwork, and autonomous practices, dimensions addressed by PPAPEP, can indirectly influence outcome indicators, which are not routinely monitored in practice settings, while promoting greater professional satisfaction, engagement, and care quality [20]. Nevertheless, in line with the structure subscale findings, organizational support and committed management are essential to observe more visible effects in this subscale.

Interestingly, the CG also showed significant improvements in the structure subscale and trends toward improvement in the outcome subscale, possibly reflecting a regression‐to‐the‐mean effect or external factors that influenced perceptions during the study period. These findings suggest that although PPAPEP may have contributed to a more positive perception of the nursing practice environment, its immediate impact was limited. The high correlations between pre‐ and postintervention values in both groups indicate measurement consistency and reinforce the need to interpret small effects cautiously. Follow‐up analyses revealed an inverted U‐shaped pattern in the IG, with an immediate increase after the intervention followed by a slight decrease at 3 months. While this decrease was not statistically significant, polynomial contrast analysis revealed a significant quadratic trend, highlighting the transient nature of perceived improvements and suggesting that sustained or repeated interventions may be necessary to maintain positive changes in practice environment perceptions.

Perceptions of the safety climate, assessed by the SAQ‐SF, remained stable in the IG, with minimal change observed between the posttest and follow‐up. By contrast, the CG showed a significant increase, suggesting that PPAPEP had no measurable short‐ or medium‐term effect on nurses’ safety climate perceptions. The stability observed in the IG may reflect a consolidated organizational culture, suggesting that brief interventions alone may be insufficient to modify safety attitudes.

The study by Finn et al. [25] also found that long‐term interventions result in gradual acquisition of changes, whereas short‐term interventions produce only minor changes. Leadership support was also emphasized as a key factor associated with strong institutional support [25]. Similarly, Amiri et al. [26] attributed the lack of effect of their adult ICU education program to the absence of manager and leader involvement. These findings underscore the need for continuous strategies or periodic reinforcement to sustain improvements in the work environment and suggest that future studies with larger samples, multiple groups, and prolonged interventions may provide more robust evidence on intervention effects.

The SEE‐NP correlated strongly with the SAQ at all time points, suggesting a strong association between perceptions of the work environment and safety attitudes and demonstrating that positive work environment perceptions are strongly associated with more favorable safety attitudes. Several studies have also found a correlation between safety perception and the nursing work environment, showing that safety perception is strongly linked to nurse support, managerial capacity, and leadership, concluding that institutions should promote leadership that prioritizes safety, encourages collaborative work, and incorporates nurse feedback in continuous improvement processes [7–9, 27, 28]. Similarly, El‐Sayed et al. [29] emphasize this relationship, identifying the nursing practice environment as a factor that strengthens patient safety and highlighting the importance of the work environment in encouraging error reporting.

When compared with established international frameworks such as the Magnet Recognition Program or the Healthy Work Environment (HWE) standards, PPAPEP offers a complementary and context‐specific approach. While Magnet and HWE models emphasize large‐scale organizational accreditation and structural transformation, PPAPEP is designed as a participatory, nurse‐centered educational intervention. Its focus on professional empowerment, communication, and teamwork supports scalability and implementation in settings where formal accreditation processes may be less feasible. In this sense, PPAPEP advances existing models by offering a pragmatic, low‐cost strategy that can act as a catalyst for broader organizational change.

Correlations between the two scales also increased over time, suggesting not only stability in perceptions, but also that perceptions of safety and the professional environment became more interdependent over time, indicating that sustained improvements in the institutional environment may strengthen a safety culture. Lucas et al. [30] conducted a comprehensive descriptive study in primary healthcare units, finding that the quality of the nursing practice environment is associated with higher quality and safer care, indicating that improving nurses’ work environment is a low‐cost organizational strategy to achieve better patient outcomes.

Nurses’ perceptions of the work environment and safety‐related attitudes are strongly interlinked and influenced by contextual and professional factors, particularly workplace and professional category. The workplace showed negative correlations with both instruments, suggesting that nurses in less favorable or high‐pressure contexts perceive the environment and safety culture more negatively. Professional category also influenced perceptions, with specialist or higher‐ranking nurses reporting more critical assessments of the environment and safety attitudes. However, variables such as age, sex, marital status, academic qualifications, years of professional practice, time in primary healthcare, and contract type did not significantly correlate with any outcomes, suggesting these factors do not substantially influence environment or safety perceptions.

The findings highlight the complexity of modifying nurses’ perceptions of the work environment and safety climate. Although PPAPEP was associated with modest and short‐term improvements in practice environment perceptions, sustained gains may require institutional interventions aimed at improving safety culture, considering the organizational environment as a whole, emphasizing institutional structure, leadership, professional support, and organizational equity. Furthermore, the limited effect on the safety climate underscores the need for interventions specifically targeting attitudes and behaviors related to safety, necessitating long‐term interventions or reinforced programs implemented over time.

### 4.1. Study Limitations

Despite the study’s strengths, such as its randomized and double‐blind design and the use of validated instruments, some methodological limitations must be acknowledged. Firstly, the relatively small sample size may have limited the statistical power to detect differences of small differences, thereby increasing the risk of false‐negative results. In addition, the follow‐up period of only 3 months restricted the ability to assess the sustainability of the intervention’s effects in the medium and long term, as more lasting changes may only become evident over extended periods. The dropout rate observed in the CG during the follow‐up period further exacerbated this limitation by reducing the comparability between groups and compromising the robustness of the conclusions.

Another important limitation relates to the reliance on self‐reported instruments which may have introduced social desirability bias, as participants might have provided responses aligned with perceived expectations. Furthermore, the absence of objective indicators—such as absenteeism rates, turnover, or adverse event data—limits the ability to directly link perceived improvements in the practice environment to measurable patient safety outcomes.

It is also not possible to rule out the influence of contextual factors external to the intervention, such as organizational changes, team dynamics, or institutional policies, which may have shaped the participants’ perceptions, particularly those in the CG. These factors may have acted as potential confounders during the study period.

Although PPAPEP demonstrated short‐term effects on key dimensions of the nursing practice environment, the duration of the intervention and the three‐month follow‐up period may have been insufficient to generate sustained changes in deeply embedded organizational constructs, such as safety culture. Previous literature suggests that long‐term and continuous interventions, combined with leadership engagement, are required to consolidate cultural transformation in healthcare organizations.

Finally, it is important to note that this study was conducted exclusively with primary healthcare nurses. This group population is often neglected in interventional research, which, while making the study more relevant, may limit the generalizability of the results to other nursing practice contexts, such as hospitals or long‐term care facilities.

Future studies should be conducted with larger samples, longer follow‐up periods, and objective organizational and clinical indicators to strengthen causal inferences and enhance the robustness of outcome assessment. Additionally, strategies to mitigate participants’ dropout should be considered in order to strengthen both the internal and external validity of the results, enabling a more comprehensive understanding of the impact of PPAPEP in promoting positive nursing practice environments and patient safety.

### 4.2. Implications of the Study Findings

The results of this study have relevant implications for nursing practice, healthcare management, and future research. The modest yet positive improvements observed in nurses’ perceptions of the practice environment following the implementation of the PPAPEP suggest that targeted interventions can contribute to improving the structural and process aspects of professional practice, even in primary healthcare settings with limited resources. Although the effects were not statistically robust across all domains, the identified trends highlight the potential of structured and participatory programs to improve working conditions and, consequently, patient safety outcomes.

The limited impact of the intervention on the safety climate highlights the complexity of this construct. This concept is influenced not only by individual perceptions and professional interactions but also by organizational culture, leadership, and systemic factors that may extend beyond the scope of a single intervention, emphasizing the need for strategies that integrate individual, team, and organizational approaches to foster sustainable cultural change.

The strong associations identified between perceptions of the practice environment and safety climate reinforce the interdependence of these domains. Improving one without addressing the other may lead to limited results, making it essential that interventions are designed to simultaneously strengthen the nursing practice environment and cultivate patient safety, ensuring alignment between organizational policies, team functioning, and professional well‐being.

From a health policy perspective, the study highlights the need for policymakers and health system leaders to prioritize investment in positive nursing practice environments as a strategic pathway to improving both professional satisfaction and quality of care. For managers, the results highlight the importance of continuous reinforcement mechanisms and the contextual adaptation of interventions to ensure the sustainability of improvements over time.

Finally, the results point to relevant avenues for future research, including replicating the intervention with larger and more diverse samples, evaluating the long‐term effects, and integrating objective patient safety indicators alongside nurses’ perceptions. Such efforts are crucial in strengthening the evidence base on how structured programs can significantly transform the nursing practice environment and improve safety in primary healthcare and other care delivery contexts.

## 5. Conclusions

The PPAPEP program was found to have the capacity to promote modest improvements in nurses’ perceptions of the nursing practice environment, particularly with regard to changes in the dimensions of structure and process. However, the effects on the safety climate were minimal, suggesting that attitudes and perceptions related to patient safety are less susceptible to short‐term changes and require more targeted and sustained interventions over time. The maintenance and consolidation of the observed improvements therefore appear to depend on continuous reinforcement strategies, including periodic capacity‐building actions, supervision, and organizational support, as well as approaches that integrate individual, team, and structural factors. The strong correlation between nurses’ perceptions of the nursing practice environment and their perceived safety climate shows that improvements in one may positively influence the other. This finding reinforces the need for integrated interventions supported by leadership engagement, strategic alignment, and institutional commitment to simultaneously promote favorable working conditions and a robust safety culture.

## Funding

This work was carried out with the support of the Coordination for the Improvement of Higher Education Personnel – Brazil (CAPES) – FundingCode 001.

## Disclosure

The funders had no role in the design, conduct, or reporting of the study. This manuscript will contribute toward a PhD in Nursing Sciences for author SP.

## Ethics Statement

This study was approved by the Health Ethics Committee (CES no. 57_2024).

## Conflicts of Interest

The authors declare no conflicts of interest.

## Supporting Information

Supporting Materials. The CONSORT checklist for reporting randomized controlled trials is provided as supporting material (File S1).

## Supporting information


**Supporting Information** Additional supporting information can be found online in the Supporting Information section.

## Data Availability

The data that support the findings of this study are available from the corresponding author upon reasonable request.
